# Assessing Knowledge, Acceptance, and Anticipated Impact of Telepathology in Saudi Arabia: Insights From Healthcare Workers and Patients

**DOI:** 10.7759/cureus.49218

**Published:** 2023-11-22

**Authors:** Khaldoon Aljerian, Amira Alrashedi, Reem Alkulaibi, Razan Alsuwailem, Alhanouf Alshahrani, Ftoon M Alzahrani, Norah Alqazlan, Mohamad-Hani Temsah

**Affiliations:** 1 Department of Pathology, College of Medicine, King Saud University, Riyadh, SAU; 2 College of Medicine, King Saud University, Riyadh, SAU; 3 College of Medicine, King Saud Medical City, Riyadh, SAU; 4 Pediatric Intensive Care Unit, Pediatric Department, King Saud University Medical City, College of Medicine, King Saud University, Riyadh, SAU; 5 Evidence-Based Health Care & Knowledge Translation Research, King Saud University, Riyadh, SAU

**Keywords:** medical technology adoption, telehealth transformation in saudi arabia, digital pathology insights, modern diagnostic infrastructure, histo-telepathology, telemedicine services, e-health acceptance rates, medical innovation, healthcare digitalization, telepathology advancements

## Abstract

Introduction: Telepathology, a rapidly evolving field in modern healthcare, has the potential to significantly impact the diagnosis and management of diseases. This study aimed to assess the prevalence of acceptance and knowledge, the likelihood of future use, and the perceived advantages and disadvantages of telepathology among healthcare workers (HCWs) and patients in Saudi Arabia.

Methods: A cross-sectional study was conducted with 388 participants, including 179 HCWs and 209 adult patients across Saudi Arabia. Data were collected using a bilingual, self-administered, and anonymous computer-based questionnaire. The Statistical Package for Social Sciences (SPSS, IBM Corp., Armonk, NY) was employed for data analysis.

Results: The participants had an average knowledge rate of 80.3% (n=312) concerning telepathology. A substantial 88.16% (n=342) were inclined towards its acceptance, and an optimistic 89.97% (n=349) foresaw its potential utility. Among the respondents, 70% (n=272) pinpointed "expedited results" as the principal merit of telepathology. However, 60% (n=233) flagged "the necessity for costly infrastructure" as its chief limitation.

Conclusions: The insights derived underscore a prominent knowledge and endorsement of telepathology among the Saudi population and HCWs. Despite certain drawbacks, participants believe that telepathology is feasible and offers numerous benefits that could greatly enhance the healthcare system in Saudi Arabia. Future research should focus on evaluating its practical implementation and efficacy within healthcare facilities.

## Introduction

Over the past decade, the healthcare system has experienced a significant surge in technological advancements, leading to enhanced efficiency and improved patient care [1]. Telepathology has the potential to mitigate delays in histological diagnosis, thus reducing complications, morbidity, mortality, and associated costs while enabling earlier and more effective treatment [2,3]. Patients who live in remote areas could be more susceptible to such complications, and telemedicine could offer both faster results and a more accurate diagnosis in such cases.

Telepathology is a subset of telemedicine and pathology that incorporates telecommunication tools to enable the sharing of image-intensive pathology information across distant places for diagnostic, educational, or research purposes [4]. The limited availability of pathologists in some areas and the demand for pathologists with expertise in subspecialties have prompted many institutions to use telepathology [5]. In this approach, the glass slide and the pathologist are not in the same place; a digital image of the captured slide is displayed on the pathologist’s computer or phone screen so they can make the diagnosis [6].

There are many advantages to using telepathology, especially since we are now capable of advancing artificial intelligence (AI) and machine learning in the discipline of anatomic pathology, similar to what was limited to radiology and cardiology [7-9]. Digital images can aid not only in the diagnosis but also in detecting prognostic features without molecular testing [10]. Ultimately, there's potential for computer-aided pathology to create individualized diagnostic and treatment plans for patients [11].

Pathologists are open to the use of telepathology for educational and academic purposes, as well as for consultation and medicolegal purposes [12]. Telepathology allows rapid transformation of data [12] and enhances the quality of healthcare [13]. Despite all these benefits, a few drawbacks have been reported in the literature, including higher costs [12] and IT support issues [14].

According to previous research on telemedicine in the Kingdom of Saudi Arabia (KSA), most healthcare workers (HCWs) agree that their colleagues are willing to implement telemedicine [15]; however, we have identified no studies in the KSA exploring HCWs and patients’ acceptance of telepathology. We hypothesized that both accept telepathology. It is important to explore patients’ views and concerns about this new technology, as well as those of HCWs. Patients’ perspectives can contribute to decision-makers' judgments, as well as the ethical concerns that may arise and the literature. Therefore, in the present study, we aim to measure HCWs’ and patients’ acceptance and knowledge of telepathology and its future use in Saudi Arabia.

## Materials and methods

A cross-sectional study (online survey) was distributed among patients and HCWs in outpatient clinics at governmental and private hospitals in KSA. The self-administered questionnaire (Appendix) was distributed via a survey link that was posted on different social media platforms. The study was conducted from September 1, 2022, to November 1, 2022. The estimated minimum sample size to measure the prevalence of acceptance was 384 subjects, with a confidence interval of 95% and a 5% margin of error. The prevalence was initially set at 50% in the calculation due to the lack of studies on the views of patients and HCWs in KSA on telepathology. The questionnaire was sent to 50% more individuals than the calculated minimum sample size to reduce nonresponse bias, and a pilot study was conducted with 10% of the target sample size to evaluate the questionnaire.

The study included participants who were either patients aged 18 and above residing in Saudi Arabia or healthcare workers employed in either private or government hospitals in Saudi Arabia during the data collection period. Patients who were under 18 years old or who lived abroad and HCWs who worked outside KSA at the time of data collection were excluded. A convenient non-probability sampling technique was adopted in this study. Data were collected using an online questionnaire (Appendix), which was distributed to possible participants based on the inclusion criteria through social media platforms. The questionnaire was developed based on a previous study [12].

The first section of the questionnaire collected demographic data, including gender, age, educational level, use of technology in daily life (laptop/smartphone/smartwatch), close relatives who have had a biopsy taken, current employment status, HCW category of specialty, and years practicing medicine related to pathology. The second section consisted of closed questions; participants responded using a 4-point Likert scale. These questions addressed knowledge measures, acceptance measures, future measures, advantages, and disadvantages. The Technology Acceptance Model (TAM) was adopted in this study [16,17].

The study utilized a descriptive analysis approach, including frequencies and percentages, to analyze demographic information, measure the average level of acceptance and knowledge, and determine the advantages and disadvantages of telepathology among healthcare workers and outpatients in KSA. The future use of telepathology was also discussed. Categorical data were compared using the chi-square test. The statistical software used for the analyses was Statistical Package for the Social Sciences (SPSS) version 24.0 (IBM Corp., Armonk, NY).

Institutional review board approval statement

Ethical approval from the IRB at KSU was obtained prior to data collection (Approval E-22-7065_F18_CMED). Participants joined voluntarily without rewards, and their personal data was kept confidential. Informed consent was incorporated at the beginning of the survey.

Data sharing statement

The data utilized to substantiate the findings of this study are comprehensively included within the article itself. Raw data that support the findings of this study are available from the principal author (KA) upon reasonable request.

## Results

Table 1 shows the characteristics of the study population, which consisted of a total of 388 respondents, of which 65.7% were female and 34.3% were male. The largest group of participants (36.6%) were under 23 years old. More than half of the participants (58.5%) had a bachelor’s degree. Almost all participants (95.6%) reported “always” using technology in their daily lives. Furthermore, most participants (66.0%) had never undergone a biopsy, nor had any of their relatives. The largest group of the study population was currently working as healthcare professionals (43.0%), while 41.8% had other roles.

**Table 1:** Demographic characteristic of participants (N=388).

Demographic data		N	%
Gender	Male	133	34.3
	Female	255	65.7
Age	Less than 23 years	142	36.6
	23 to 30 years	111	28.6
	More than 30 years	135	34.8
Your educational level	High school or less	82	21.1
	Bachelor’s degree	227	58.5
	Master’s degree	24	6.2
	PhD or equivalent	55	14.2
Use technology in your daily life (laptop-smartphone-smartwatch)	Rarely	1	0.3
	Sometimes	16	4.1
	Always	371	95.6
Relatives had a biopsy taken before	Yes	132	34.0
Current role	Pathologist	12	3.1
	Healthcare professional	167	43.0
	Non-healthcare worker	47	12.1
	Other	162	41.8
Specialty falls in which category from the following	Specialty highly dependent on (biopsy, cytology, and resection)	23	13.8
	Specialty rarely dependent on tissue pathology	77	46.1
	Specialty doesn’t have any resection, biopsy, or cytology	67	40.1
Years of practicing medicine involved pathology	Up to five years	2	16.7
	6 to 10 years	3	25.0
	11 to 20 years	3	25.0
	More than 20 years	4	33.3

Table 2 shows the distribution of study participants based on their responses regarding knowledge measures. Participants’ average knowledge score was 80.30%. The table further illustrates that the highest rate of agreement (92.60%) was observed for the statement “The implementation of telepathology would facilitate hospitals in remote areas.” This indicates that the majority of participants agreed that telepathology could positively impact healthcare in remote areas.

**Table 2:** Telepathology knowledge measures of health workers and outpatients in Saudi Arabia.

Knowledge measures		N (%)			P-value
		Healthcare workers	Patients	Total	
I have a genuine understanding of telepathology	Disagree	64(16.50)	94(24.20)	158(40.70)	0.065
	Agree	115(29.60)	115(29.60)	230(59.30)	
Telepathology can be integrated with laboratory information systems	Disagree	17(4.40)	26(6.70)	43(11.10)	0.357
	Agree	162(41.80)	183(47.20)	345(89.00)	
Telepathology enables hospitals in remote areas	Disagree	9(2.30)	20 (5.20)	29(7.50)	0.090
	Agree	170(43.80)	189(48.70)	359(92.60)	
Average participant knowledge of telepathology					80.30%

Table 3 shows the distribution of study participants’ responses regarding their acceptance of measures of telepathology. The average acceptance of telepathology among participants was 88.16, according to “agree” answers to acceptance measures. Notably, 96.9% of participants agreed that adopting telepathology in hospitals in Saudi Arabia would benefit patient care, as demonstrated by the weighted number of “agree” responses for participants’ overall perception of acceptance measures.

**Table 3:** Telepathology acceptance measures of health workers and outpatients in Saudi Arabia.

Acceptance measures		N (%)			P-value
		Healthcare workers	Patients	Total	(2-sided)
Telepathology will be beneficial to patient care in hospitals at KSA	Disagree	5(1.30)	7(1.80)	12(3.10)	0.752
	Agree	174(44.90)	202(52.10)	376(96.90)	
The advantages of telepathology will outweigh the disadvantages	Disagree	21(5.40)	24(6.20)	45(11.60)	0.939
	Agree	158(40.70)	185(47.70)	343(88.40)	
The use of telepathology will be more effective than routine pathology	Disagree	25(6.50)	40(10.30)	65(16.80)	0.174
	Agree	154(39.70)	169(43.50)	323(83.20)	
It would be easy for workers in the lab to follow the instructions and operate the equipment for telepathology	Disagree	31(8.00)	52(13.40)	83(21.40)	0.070
	Agree	148(38.10)	157(40.50)	305(78.60)	
Using telepathology enables the pathologist to have accurate information	Disagree	24(6.30)	25(6.50)	49(12.60)	0.669
	Agree	155(39.90)	184(47.40)	339(87.30)	
I would accept being diagnosed/making a diagnosis using telepathology	Disagree	21(5.40)	29(7.50)	50(12.90)	0.530
	Agree	158(40.70)	180(46.40)	338(87.10)	
The adaptation of telepathology would reduce the risks related to health	Disagree	18(4.70)	25(6.40)	43(11.10)	0.551
	Agree	161(41.50)	184(47.70)	345(88.90)	
I Am glad to present telepathology to my closest ones	Disagree	12(3.10)	13(3.40)	25(6.40)	0.847
	Agree	167(43.00)	196(50.50)	363(93.60)	
I will adopt telepathology based on my close one's necessities	Disagree	19(4.90)	22(5.70)	41(10.60)	0.974
	Agree	160(41.20)	187(48.20)	347(89.40)	
Average participant acceptance of telepathology					88.16%

Participants’ perceptions about the future of telepathology are shown in Table 4. According to average “agree” in future measures, participants were 89.97% believing that telepathology will be applied in the future. Participants were asked about their views on various future measures; the statement that participants agreed with most was “I believe telepathology will be helpful during lockdowns such as COVID-19,” with a weighted agreement percentage of 94.30%.

**Table 4:** Telepathology Future measures of health workers and outpatient in Saudi Arabia.

Future measure’s		N (%)			P value
		Healthcare workers	Patients	Total	(2-sided)
Virtual microscopy will be the golden standard in the future	Disagree	32(8.30)	40(10.30)	72(18.50)	0.750
	Agree	147(37.90)	169(43.60)	316(81.50)	
Telepathology will boost computer-aided diagnosis	Disagree	10(2.60)	13(3.40)	23(5.90)	0.792
	Agree	169(43.50)	196(50.50)	365(94.10)	
I believe telepathology will be helpful during lockdowns such as COVID-19	Disagree	8(2.10)	14(3.60)	22(5.70)	0.344
	Agree	171(44.40)	195(50.30)	366(94.30)	
Average of participant believes in the future application of telepathology				89.97%	

**Figure 1:**
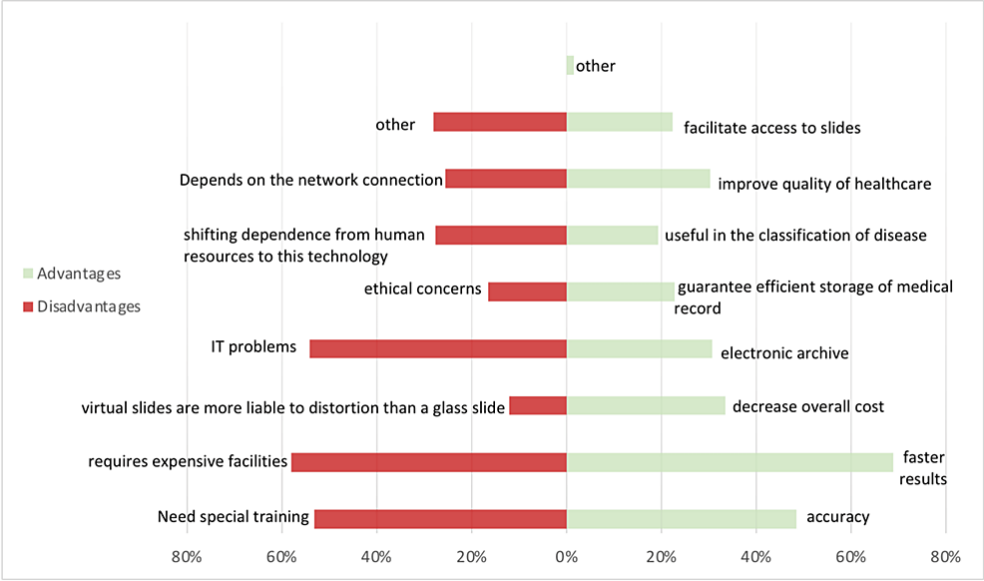
Advantages and disadvantages percentage according to respondents.

Figure 1 shows the distribution of study participants’ responses regarding the advantages and disadvantages of telepathology. Most participants (68.8%) believed that the most significant advantage of telepathology was faster results. The most frequently chosen disadvantage of telepathology, selected by 58.0% of participants, was that it requires expensive facilities.

## Discussion

These results show that HCWs and patients in outpatient clinics are aware of telepathology; we also found no significant difference in HCWs’ and patients’ awareness of telepathology (p > 0.05). In contrast, Gozali et al. report significant differences in awareness of telepathology among pathologists, information technology (IT) professionals, hospital managers, and directors [13]. These differences could be due to the use of different questionnaires targeting different populations as well as increases in knowledge of technology in recent years, which could have decreased the “knowledge gap” between HCWs and patients.

We found that 80.30% of participants knew about telepathology, which is consistent with Mairinger, who reported a high level of knowledge and information regarding telepathology among HCWs in the field of pathology [18]. It is possible that the high level of knowledge in our study is due to the current high level of awareness about technology; in the previous study, the high level of knowledge could be attributed to the participant population, as this study targeted professionals in the field. We also found high acceptance of telepathology among HCWs and outpatients (88.16%). This is similar to other studies finding high levels of acceptance among pathologists [19,20]. For example, Chordia et al. report that 98% of pathologists consider telepathology essential [12]. These studies show high levels of acceptance, which could be due to a high awareness of the importance of telepathology.

In terms of future measures, the participants in our study strongly agreed with the potential usefulness of telepathology during lockdowns, such as those implemented during the COVID-19 pandemic, with a weighted agreement percentage of 94.30%. This finding is consistent with Koelzer et al., who reported that 56.8% of respondents considered telepathology useful during the COVID-19 pandemic [21]. Similarly, Chordia et al. reported that 51% of participants agreed that telepathology would be frequently used and recommended over the next five years [12]. It is important to note that this prediction of future usage includes all cases and not just pandemics. Furthermore, we found that 94.10% of participants believed that telepathology would enhance computer-aided diagnoses. This is consistent with Unternaehrer et al.; in that study, 82% of respondents indicated that computer-based diagnostics would facilitate their work [14].

There are different opinions about the advantages and disadvantages of telepathology. Our results showed the most important advantage of telepathology was faster results; 68.8% of our participants highlighted this advantage, in line with Chordia et al. In light of recent geographical expansions, rapid development, and population growth, hospitals in Saudi Arabia need such technology and the faster results it can provide. where, according to Unternaehrer et al., participants supported the use of telepathology to obtain a second opinion [14]. The most chosen disadvantage of telepathology was the need for expensive facilities; 58% of our participants highlighted this issue, which is also in line with Chordia et al. In contrast, Unternaehrer et al. identified IT problems as the most important disadvantage [14].

Our study is the first to highlight the significant benefits that telepathology could offer the Saudi healthcare system. Still, the study has some limitations that should be taken into consideration when interpreting the results, including the possibility of selection bias due to the use of a self-administered questionnaire. We recommend that future studies explore the knowledge, acceptance, and future applications of telepathology among pathologists. A noteworthy development in the realm of artificial intelligence is the Chat Generative Pre-trained Transformer (ChatGPT), which has recently acquired the capability to interpret images and discern patterns and is rapidly evolving in the medical fields [22,23]. Its potential role as a "telepathology advisor" remains a potential prospect, warranting exploration in forthcoming research. In addition, future similar studies could describe participants from multiple areas across Saudi Arabia, especially remote healthcare centers. Future research might also be conducted after telepathology has been implemented in some hospitals to evaluate its efficacy, efficiency, accuracy, and overall cost-effectiveness. It is important to note that most participants (66.0%) had never undergone a biopsy, nor had any of their relatives, which may have influenced their responses to the questions. The participants' lack of experience in whole slide imaging was also acknowledged, and this is an area that future studies could explore further.

## Conclusions

In conclusion, the majority of participants in this study, encompassing both HCWs and patients, exhibited substantial knowledge and a high level of acceptance toward telepathology. Despite the acknowledged disadvantages, participants expressed their belief in the feasibility, potential success, and overall benefits of introducing more telepathology into the Saudi Arabian healthcare setting. This optimism regarding the positive impact on the healthcare system suggests that the integration of telepathology could lead to enhanced patient outcomes and greater resilience in the healthcare sector. Future research may explore strategies for optimizing the implementation of telepathology in healthcare settings.

## Appendices

Questionnaire: acceptance of telepathology among healthcare workers and visitors to outpatient clinics

We are a group of students from the College of Medicine at KSU, under the supervision of Dr. Khaldoon Aljerian, pathology and forensics department, working on research to measure acceptance among healthcare workers and outpatient clinics. The questionnaire will take around three minutes of your time and has received IRB approval from KSU. The responses are completely anonymous and confidential. We will use this collected information for research purposes only. Completing the survey indicates your consent to participate. Thank you for your valuable input.

**Table 5:** Questionnaire section A: Socio-demographic

Questionnaire Section A: Socio-demographic								
1-Gender:				Female			Male	
2-Age (please write down)				Years				
3-Your educational level				High school or less	Bachelor’s degree		master’s degree	Ph.D. or equivalent
4-How often do you use technology in your daily life (laptop, smartphone or smartwatch)?				Always	Sometimes		Rarely	Never
5-Have you or any of your close relatives had a biopsy taken before?				Yes			No	
6- What is your current role?				Pathologist	Healthcare professional		Non- healthcare worker	Other (please specify)
7-If you are a healthcare worker, you feel your specialty falls in which category from the following:								
A. Specialty highly dependent on (biopsy, cytology, resection)		B. Specialty rarely dependent on tissue pathology			c. Specialty doesn’t have any resection, biopsy, or cytology			
8-If you are a pathologist, how many years have you been practicing medicine involved with pathology?								
A. Up to five years	B. 6 to 10 years		C. 11 to 20 years			D. more than 20 years		

**Table 6:** Questionnaire section B: Telepathology knowledge measures

B: Knowledge measures: rate your level of agreement with each statement	Strongly disagree	Disagree	Agree	Strongly agree
9. I have a genuine understanding of Telepathology	1	2	3	4
10. Telepathology can be integrated into laboratory information systems	1	2	3	4
11. Telepathology enables hospitals in remote areas	1	2	3	4

**Table 7:** Questionnaire section C: Telepothology acceptance measures

C: Acceptance measures: rate your level of agreement with each statement	Strongly disagree	Disagree	Agree	Strongly agree
12. Telepathology will be beneficial to patient care in hospitals at KSA	1	2	3	4
13. The advantages of telepathology will outweigh the disadvantages	1	2	3	4
14. The use of telepathology will be more effective than routine pathology	1	2	3	4
15. It would be easy for workers in the lab to follow the instructions and operate the equipment for telepathology	1	2	3	4
16. Using telepathology enables the pathologist to have accurate information	1	2	3	4
17. I would accept being diagnosed/making a diagnosis using telepathology	1	2	3	4
18. The adaptation of telepathology would reduce the risks related to health	1	2	3	4
19. I Am glad to present telepathology to my closest ones	1	2	3	4
20. I will adopt telepathology based on my close ones' necessities	1	2	3	4

**Table 8:** Questionnaire section D: Future measures

D: Future measures: Rate your level of agreement with each statement	Strongly disagree	Disagree	Agree	Strongly agree
21. Virtual microscopy will be the golden standard in the future	1	2	3	4
22. Telepathology will boost computer-aided diagnosis	1	2	3	4
23. I believe Telepathology will be helpful during lockdowns such as COVID-19	1	2	3	4

Section E: advantages

24  -In your opinion what are the most important advantages of telepathology (choose up to 3)

a.   -Accuracy

b.   -Faster results

c.   -Decrease overall cost

d.   -Electronic archive

e.   -Guarantee efficient storage of medical records

f.    -Useful in classification of disease

g.   -Improve quality of healthcare

h.   -Facilitate access to slides

i.    -Other (please specify)

Section F: disadvantages

25   -In your opinion what are the most important disadvantages of Telepathology?

(Choose up to 3)

a.    -Need special training

b.    -Requires expensive facilities

c.    -Virtual slides are more liable to distortion than a glass slide

d.    -IT problems

e.    -Ethical concerns

f.     -Shifting dependence from human resources to this technology

g.    -Depends on network connection

h.    -Other (please specify)
